# Gigahertz single-trap electron pumps in silicon

**DOI:** 10.1038/ncomms6038

**Published:** 2014-10-06

**Authors:** Gento Yamahata, Katsuhiko Nishiguchi, Akira Fujiwara

**Affiliations:** 1 NTT Basic Research Laboratories, NTT Corporation, 3-1 Morinosato Wakamiya, Atsugi, Kanagawa 243-0198, Japan

## Abstract

Manipulation of single electrons is the key to developing ultimate electronics such as single-electron-based information processors and electrical standards in metrology. Especially, high-frequency and high-accuracy single-electron pumps are essential to realize practical current standards. While electrically defined quantum dots are widely used to build single-electron pumps, a localized state in semiconductors is also a potential candidate for accurate pumps because it can have a large activation energy for the captured electron. However, the transfer mechanism of such localized-state-mediated single-electron pumps for high-accuracy operation at a high frequency has not been well examined. Here we demonstrate a single-electron pump using a single-trap level with an activation energy of a few ten millielectron volts in Si nanotransistors. By means of gate control of capture and emission rates, the pump operates at a frequency of 3 GHz with an accuracy of better than 10^−3^ at 17 K, indicating that an electric field at the trap level lowers the capture and emission time to less than 25 ps.

## Abstract (web summary)

Devices that generate single electrons are crucial for a precise definition of the ampere—the SI unit for electrical current. Yamahata *et al*. now demonstrate a silicon-based transistor that can accurately emit lone electrons at a rate of over 3 billion per second.

## Graphical abstract

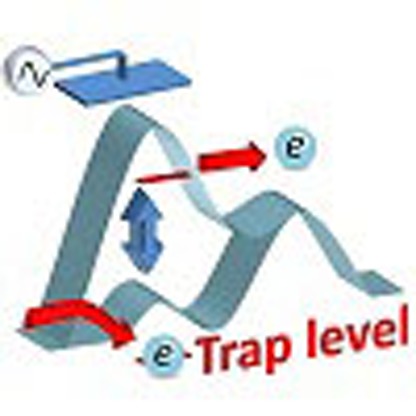

A single-electron (SE) pump enables clocked transfer of an integer number of the elementary charge *e*, which can be used for realizing SE circuits^1^, coherent SE sources^2^ and metrological current standards^3^. In particular, the current standards have attracted much attention because they are directly linked to a redefinition of the SI unit of electric current (the ampere)^3^,^4^ by fixing *e*. To realize practical current standards, a relative error rate of less than 10^−8^ and a current level of more than several hundred picoamperes (gigahertz operation) are indispensable^5^. A sufficiently low error rate has been reported^6^ in metal-based SE pumps^7^,^8^,^9^, but the operating speed is low because of the fixed tunnel barriers with high resistances. In contrast, semiconductor-based SE pumps using electrically defined islands in Si^10^,^11^,^12^,^13^,^14^,^15^,^16^ or GaAs^17^,^18^,^19^,^20^,^21^ have been operated at a sufficiently high speed because the tunability of their tunnel barriers allows one to create low-resistance barriers. So far, the best result^21^ is 945-MHz operation (~150 pA) with an uncertainty of about 10^−6^, which is limited by the measurement systems. This was achieved in a GaAs pump, for which a low-temperature (*T*<1 K) and a high magnetic field (*B*~14 T) are required. In addition, while the theoretical prediction is that the best transfer accuracy in the GaAs SE pump^21^ as well as in an Si SE turnstile^22^ approaches the order of 10^−8^, we should further improve the device performance.

Semiconductor SE pumps using localized states, such as dopants implanted in a channel^23^,^24^,^25^, instead of electrically defined islands have been studied with increasing interest. Very recently, high-speed operation of a single donor pump has been reported^26^, demonstrating the potential of such a pump. There are two merits of the SE pumps using a localized state. The more important one is that a localized state can inherently have a large activation energy, leading to a large electron addition energy. This results in low error rates of the SE transfer. The other merit is that it is possible to increase the transfer current by introducing many localized states^24^ (a similar idea is parallel SE pumping^27^,^28^). However, a high electric field is necessary for high-accuracy operation at a high frequency because a large activation energy inevitably leads to the need for a high electric field to emit an SE from the localized state. It is not clear whether we can keep the device performance stable with such a high electric field. To address the possibility of high-frequency SE transfer with high accuracy, we utilized a trap level as the localized state. Historically, there have been many reports about trap levels in Si/SiO_2_ systems, which are detected by using several measurement techniques such as capacitance or conductance measurements^29^, measurements of random telegraph signals^30^, deep-level transient spectroscopy^31^ and charge pumping measurements^32^. From these measurements, we have acquired valuable knowledge about the trap density and capture cross section of the trap levels, but the potential for high-performance SE transfer is still uncertain.

Here, we study the transfer mechanism of an SE pump using a single-trap level in Si metal–oxide–semiconductor field-effect transistors (MOSFETs). Owing to electrical controllability of the SE capture/emission rates to/from the single-trap level, we achieved high-speed SE transfer of up to 3.5 GHz with a transfer accuracy of about 10^−3^ limited by the total measurement uncertainty. The SE pump operates at a relatively high temperature (*T*=17 K) without a magnetic field because of the large activation energy of the trap level.

## Results

### Device structure and measurement scheme

**Figure 1:**
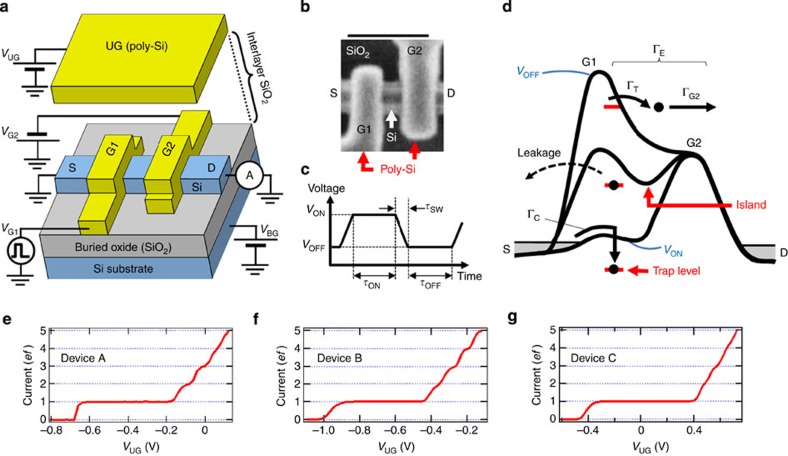
Device structure, model of the trap-mediated transfer and typical data. (**a**) Schematic of the device with the measurement configuration. (**b**) Scanning electron microscope image of the device before UG formation. The length of the scale bar is 200 nm. (**c**) Schematic of a pulse sequence for SE transfer with definitions of pulse parameters. The pulse is applied to G1. (**d**) Energy diagram with a model of the SE transfer via a trap level (trap-mediated transfer). SEs are also transferred via a charge island between G1 and G2 (island-mediated transfer). The transfer direction is from the left (S) to right (D) leads. (**e**–**g**) Typical current normalized by *ef* as a function of voltage applied to the UG (*V*_UG_) for three devices. For all devices, temperature *T*=17 K, operating frequency *f*=10 MHz, *τ*_SW_=1 ns and *τ*_ON_=*τ*_OFF_. For device A (**e**), voltage applied to G2 *V*_G2_=−0.6 V, voltage applied to the Si substrate or the backgate *V*_BG_=10 V, *V*_ON_=0.25 V and *V*_OFF_=−1.5 V. For device B (**f**), *V*_G2_=−0.65 V, *V*_BG_=20 V, *V*_ON_=0.75 V and *V*_OFF_=−1.5 V. For device C (**g**), *V*_G2_=−0.9 V, *V*_BG_=10 V, *V*_ON_=0.5 V and *V*_OFF_=−2.5 V. The 1*ef* plateaus correspond to the trap-mediated transfer as discussed in the main text.

Figure 1a,b respectively shows a schematic and a scanning electron microscope image of the device, which comprises an Si nanowire on SiO_2_ with a double-layer gate structure: two lower gates (G1, G2) and an upper gate (UG). The fabrication process is described in Methods. To transfer SEs, we modulate a single potential barrier under G1 by applying high-frequency signals to it with frequency *f* (mostly we used voltage pulses; details of the measurement systems are described in Methods)^12^. A negative voltage applied to G2 creates a fixed potential barrier under it. The UG is used to control the potential of a charge island between G1 and G2. Figure 1c shows the definitions of parameters for the voltage pulses (*V*_ON_, *V*_OFF_, *τ*_ON_, *τ*_OFF_, *τ*_SW_). We measured the SE transfer current passing from the left (S) to right (D) leads.

### Mechanism of trap-mediated transfer

When a trap level is located under G1 as depicted in an energy diagram (Fig. 1d), an SE is transferred via the trap level, which is hereafter called trap-mediated transfer. We considered a model of the trap-mediated transfer, which consists of three parts: SE capture with rate Γ_C_ to the trap level during *τ*_ON_, SE leakage to the left lead during *τ*_SW_ and SE emission with rate Γ_E_ from the trap to the right lead during *τ*_OFF_ (Γ_E_ is divided into Γ_T_ (detrap) and Γ_G2_ (escape over the barrier under G2)). Since one SE is transferred per cycle, the resultant transfer current level is 1*ef*. To perform high-speed and high-accuracy trap-mediated transfer, Γ_C_ and Γ_E_ should be much faster than 1/*τ*_ON_ and 1/*τ*_OFF_, respectively, and the SE leakage should be minimized. It can be readily speculated that, as shown in Fig. 1d, the optimal location of the trap would be a little to the right of the barrier top for directional transfer from the left to right leads. On the other hand, SE transfer via the charge island (hereafter called island-mediated transfer), in which the SE is captured to the island and then emitted with Γ_G2_ (refs 12, 22, 33, 34), is well known. The fact that island-mediated transfer does not have the detrapping process (Γ_T_) allows us to distinguish the two types of SE transfers as shown below.

We here demonstrate the SE transfer using the SE pump with a trap level. Figure 1e–g shows the current normalized by *ef* as a function of voltage applied to the UG (*V*_UG_) in three devices, where we observed clear current plateaus. By investigating three signatures of the trap-mediated transfer discussed below, we conclude that the 1*ef* plateau originates from the trap-mediated transfer, and the other plateaus (higher than 1*ef*) consist of the trap-mediated (contribution of 1*ef*) and island-mediated transfers. Note that we have tested many devices to find ones that have a trap level at the optimal position.

The first and easily visualized signature of the trap-mediated transfer is a wide current plateau, which is clearly observed in the three devices, owing to the large activation energy of the trap level. Generally speaking, the addition energy for the first electron is larger than that for the second electron even in the island because of the decrease in effective island size with decreasing number of electrons, especially in a few-electron regime^35^. However, the ratio of the width of the 1*ef* plateau to that of the 2*ef* plateau for the trap-mediated transfer (~5.6 for device A, ~7.1 for device B and ~11 for device C shown in Fig. 1e–g, respectively) is much larger than that of the island-mediated transfer in an equivalent-sized Si device that has no trap levels (~1.1 for the device shown in ref. 12). Note that the ratio is about 1.5 even in a smaller Si dot^36^.

The second signature is the tunability of the width of the 1*ef* plateau by applying backgate voltage *V*_BG_^11^, which is not observed for the device without the trap levels because application of *V*_BG_ simply shifts the threshold voltage of all plateaus of the island-mediated transfer. The width tunability most likely originates from the different spacial positions of the island and trap level (more details including the *V*_BG_ modulation for device A are described in Supplementary Note 1 and Supplementary Fig. 1).

**Figure 2:**
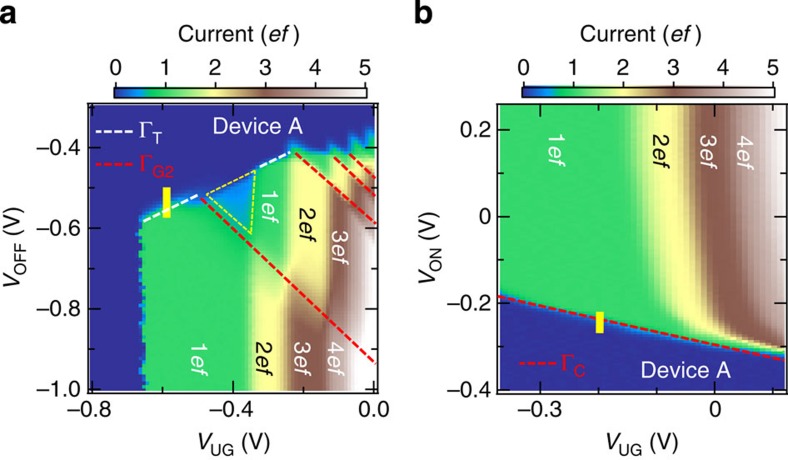
Gate-voltage dependence of capture and emission. (**a**) Two-dimensional plot of the current normalized by *ef* as a function of *V*_UG_ and *V*_OFF_ in device A, where *f*=10 MHz, *V*_G2_=−0.35 V, *V*_BG_=10 V, *V*_ON_=0.25 V, *τ*_SW_=1 ns, *τ*_ON_=*τ*_OFF_ and *T*=17 K. The positive slope of the boundary (white dashed line) strongly indicates the trap-mediated transfer as discussed in the main text. (**b**) Two-dimensional plot of the current normalized by *ef* as a function of *V*_UG_ and *V*_ON_ in device A, where *f*=10 MHz, *V*_G2_=−0.6 V, *V*_BG_=10 V, *V*_OFF_=−2 V, *τ*_SW_=1 ns, *τ*_ON_=*τ*_OFF_ and *T*=17 K. The 1*ef* plateau originates from the trap-mediated transfer and both the trap-mediated transfer and the island-mediated transfer contribute at plateaus higher than the 1*ef* plateau, which is determined from **a**.

The third and most decisive signature is revealed when we investigate the SE emission process because only the trap-mediated transfer has the detrapping process (Γ_T_). To investigate it, we measured the transfer current as a function of *V*_UG_ and *V*_OFF_ (Fig. 2a), where *V*_ON_ is sufficiently high. It is shown that the transfer current decreases with increasing *V*_OFF_ because of insufficient SE emission during *τ*_OFF_. In addition, there are boundaries having a positive slope (white dashed line) and a negative slope (red dashed line), where the difference in the current is 1*ef* vertically across these boundaries. It is also seen that the current less than 1*ef* flows in the region indicated by the yellow triangle.

The positive and negative slopes in Fig. 2a correspond to Γ_T_ and Γ_G2_, respectively, which is explained as follows. The increase in both Γ_T_ and Γ_G2_ leads to a current increase. Γ_T_ should increase when the electric field at the trap level increases, whereas Γ_G2_ should increase when the height between the top of the barrier under G2 and the island potential (hereafter called G2-to-island barrier height) decreases. When *V*_OFF_ increases, the potential barrier under G1 is lowered and thus the electric field at the trap level decreases. Simultaneously, since the island potential is also lowered because of the capacitive coupling between G1 and the island, the G2-to-island barrier height increases. As a result, both Γ_T_ and Γ_G2_ decrease. On the other hand, when *V*_UG_ increases, the island potential is lowered more than the two barrier potentials because the effect of *V*_UG_ is screened by G1 and G2. Thus, Γ_T_ increases due to the increased electric field, but Γ_G2_ decreases because of the increased G2-to-island barrier height. In this way, Γ_G2_ has the same dependence on *V*_OFF_ and *V*_UG_, but Γ_T_ depends on *V*_OFF_ and *V*_UG_ differently, which leads to the negative and positive slopes in Fig. 2a. We therefore conclude that the positive slope in the *V*_UG_−*V*_OFF_ mapping is a strong indication of the trap-mediated transfer that can be dominated by Γ_T_. Noteworthy is that the yellow triangle is the region where the trap-mediated transfer is also affected by Γ_G2_, suggesting that the SE emitted from the trap level relaxes to the bottom of the island potential with some possibility and insufficient emission due to Γ_G2_ occurs.

We then show the current normalized by *ef* as a function of *V*_UG_ and *V*_ON_ (Fig. 2b), where *V*_OFF_ is sufficiently low, to investigate the *V*_ON_ dependence. It is seen that the current decreases with decreasing *V*_ON_ because of the insufficient SE capture. A boundary having a negative slope (red dashed line) is shared among all plateaus. Since the SE capture for the island-mediated transfer during *τ*_ON_ should be determined by the height between the top of the barrier under G1 and the Fermi level of the left lead (hereafter called G1-barrier height), Γ_C_ for the trap-mediated transfer in device A should also be determined by it. The reason the slope is negative is that the G1-barrier height decreases with both increasing *V*_UG_ and *V*_ON_. Note that a different process related to the capture cross section of the trap level would appear in a different measurement condition as discussed below.

### Electrical control of capture and emission rates

**Figure 3:**
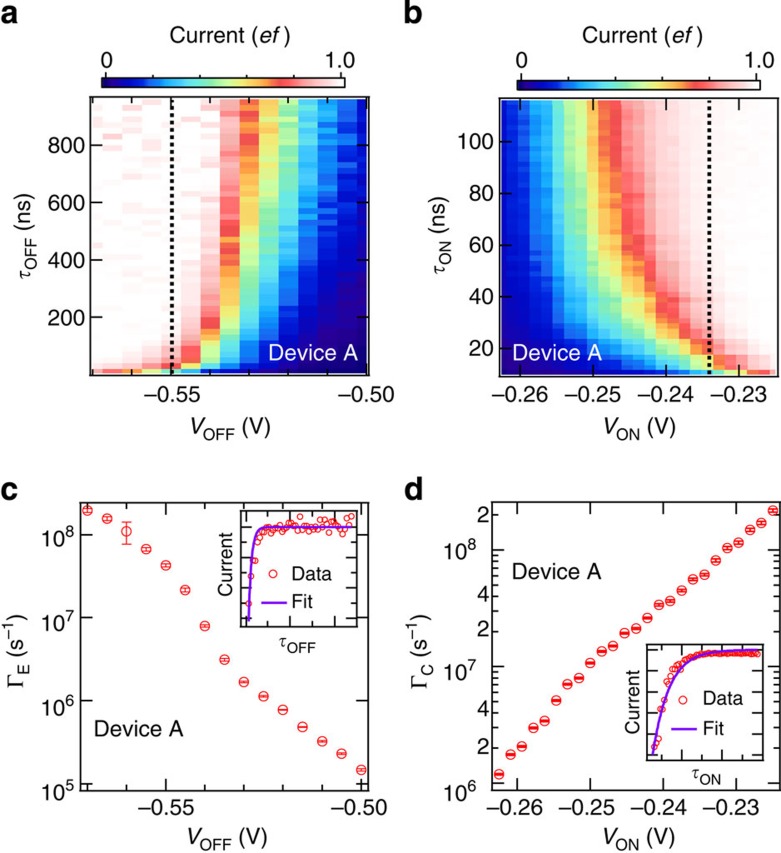
Electrical control of capture and emission rates for trap-mediated transfer. (**a**) Two-dimensional plot of the current normalized by *ef* as a function of *V*_OFF_ and *τ*_OFF_ in device A, where *V*_UG_=−0.59 V, *V*_G2_=−0.35 V, *V*_BG_=10 V, *V*_ON_=0.25 V, *τ*_SW_=1 ns, *τ*_ON_=50 ns and *T*=17 K. To keep *τ*_ON_ constant, *f* and *τ*_OFF_ were simultaneously swept. (**b**) Two-dimensional plot of the current normalized by *ef* as a function of *V*_ON_ and *τ*_ON_ in device A, where *V*_UG_=−0.2 V, *V*_G2_=−0.6 V, *V*_BG_=10 V, *V*_OFF_=−2 V, *τ*_SW_=1 ns, *τ*_OFF_=50 ns and *T*=17 K. To keep *τ*_OFF_ constant, *f* and *τ*_ON_ were simultaneously swept. (**c**) Emission rate Γ_E_ from the trap level to the right lead during the off-state of the pulse as a function of *V*_OFF_, extracted from a fit to the data in **a**, where the error bars are the s.d. of the fit. By applying large negative *V*_OFF_, the operating speed increases to more than 10^8^ s^−1^. Inset: the normalized current along with *τ*_OFF_ (red dots), corresponding to the dotted line in **a**, with a theoretical fit (purple curve). (**d**) Capture rate Γ_C_ to the trap level during the on-state of the pulse as a function of *V*_ON_, extracted from a fit to the data in **b**, where the error bars are the s.d. of the fit. By applying large positive *V*_ON_, the operating speed increases to more than 10^8^ s^−1^. Inset: the normalized current along with *τ*_ON_ (red dots), corresponding to the dotted line in **b**, with a theoretical fit (purple curve).

To investigate the electrical controllability of Γ_C_ and Γ_E_, we extracted them from time-dependence measurements using theoretical SE transfer probability *P* based on a simple exponential decay, which is given by (the derivation and more details are discussed in Supplementary Note 2)


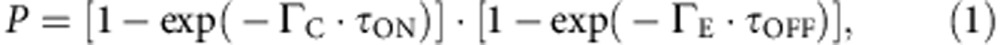


where we ignored the leakage to the left lead because we observed the 1*ef* plateau by applying sufficiently high *V*_ON_ and low *V*_OFF_. *τ*_OFF_ and *τ*_ON_ dependences measured at the yellow lines in Fig. 2a,b, respectively, are shown in Fig. 3a,b. In Fig. 3a, since Γ_C_ is much faster than 1/*τ*_ON_ because of the enormous number of charge carriers induced by applying large *V*_ON_, the SE transfer is dominated by the SE emission and therefore *P*~1−exp(−Γ_E_·*τ*_OFF_). Using this equation, we fitted the data to extract Γ_E_ (see inset in Fig. 3c). Similarly, Γ_C_ was extracted from the *τ*_ON_ dependence, where we use *P*~1−exp(−Γ_C_·*τ*_ON_) as a fit function (see inset in Fig. 3d). Figure 3c,d shows extracted Γ_E_ and Γ_C_ as a function of *V*_OFF_ and *V*_ON_, respectively. Γ_E_ and Γ_C_ increase almost exponentially up to more than 10^8^ s^−1^ with decreasing *V*_OFF_ and increasing *V*_ON_, respectively.

Next, we discuss the possibility of speeding up of the capture and emission, because the time-dependence measurements that were shorter than 10 ns were difficult to perform in our measurement systems. For the emission process, since we can apply a stronger electric field with decreasing *V*_OFF_, we can expect to achieve faster Γ_E_. In contrast, Γ_C_ should increase further with increasing *V*_ON_ because the G1-barrier height decreases further; however when the G1-barrier height is zero, the capture cross section *σ* of the trap level would limit Γ_C_. In this case, Γ_C_ should be determined by *σ*, the mean carrier velocity 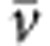 and the density of electrons in the channel *n*_*e*_, resulting in 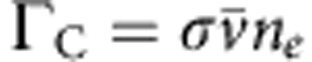 (ref. 30). We estimated Γ_C_ from typical values^29^ in the strong inversion regime: *σ*~8 × 10^−16^ cm^2^, 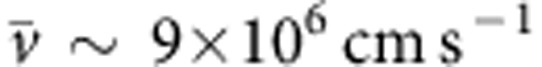, and *n*_*e*_~3 × 10^18^ cm^−3^, where we assumed a two-dimensional inversion layer with a thickness of 5 nm. The estimated Γ_C_ is about 2 × 10^10^ s^−1^, which is fast enough to achieve a few gigahertz operation. Note that this is order estimation because *σ* strongly depends on the position of the trap level.

### Activation energy

**Figure 4:**
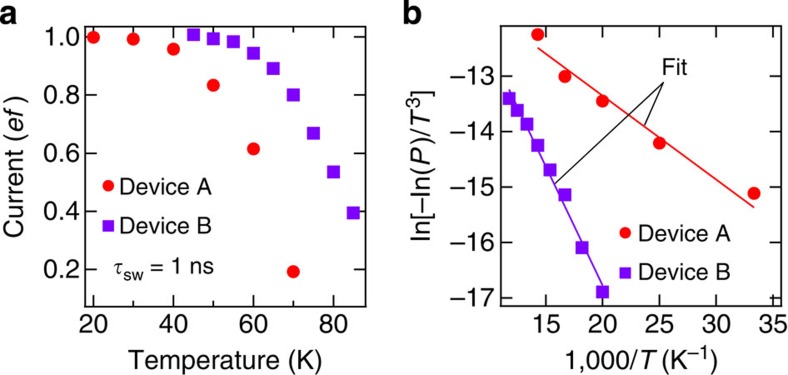
Temperature dependence of trap-mediated transfer. (**a**) Current normalized by *ef* at the centre of the 1*ef* plateau as a function of temperature in devices A and B. The measurement conditions for device A are as follows: *f*=100 MHz, *V*_UG_=−0.34 V, *V*_G2_=−0.6 V, *V*_BG_=10 V, *V*_ON_=0.25 V, *V*_OFF_=−1.5 V, *τ*_SW_=1 ns and *τ*_ON_=*τ*_ON_. The measurement conditions for device B are as follows: *f*=10 MHz, *V*_UG_=0.205 V, *V*_G2_=−1 V, *V*_BG_=5 V, *V*_ON_=0.5 V, *V*_OFF_=−2.5 V, *τ*_SW_=1 ns and *τ*_ON_=*τ*_ON_. The error bars corresponding to the total measurement uncertainties are too small to be visible in this axis scale. (**b**) ln[−ln(*P*)/*T*^3^] for devices A and B as a function of an inverse of temperature, where *P* (=the normalized current in **a**) is the transfer probability. The two lines are linear fits.

Since different traps should have different activation energies, we here estimate the activation energy of the trap level in two devices (devices A and B). Note that we verified that the 1*ef* plateau of device B is also the trap-mediated transfer by checking the slope of the boundary of the 1*ef* plateau on the *V*_UG_–*V*_OFF_ plane (the data are shown in Supplementary Fig. 2). The temperature dependence of the transfer current at the centre of the 1*ef* plateau is shown in Fig. 4a. It is seen that the current decreases with increasing temperature because the leakage to the left lead is enhanced. Since the operation temperature of device B is higher than that of device A, we readily speculate that the activation energy of device B is larger than that of device A.

To extract the activation energy, we used the theory of the thermal emission from a single level during barrier modulation^24^. The transfer probability *P* is given by


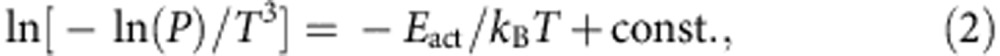


where *E*_act_ is the activation energy when the trap level aligns with the Fermi level of the lead and *k*_B_ is the Boltzmann constant. The fitting results are shown in Fig. 4b, where *E*_act_ is estimated to be about 13 and 37 meV for devices A and B, respectively. As expected, device B has a larger *E*_act_ than device A.

### High-speed trap-mediated transfer

**Figure 5:**
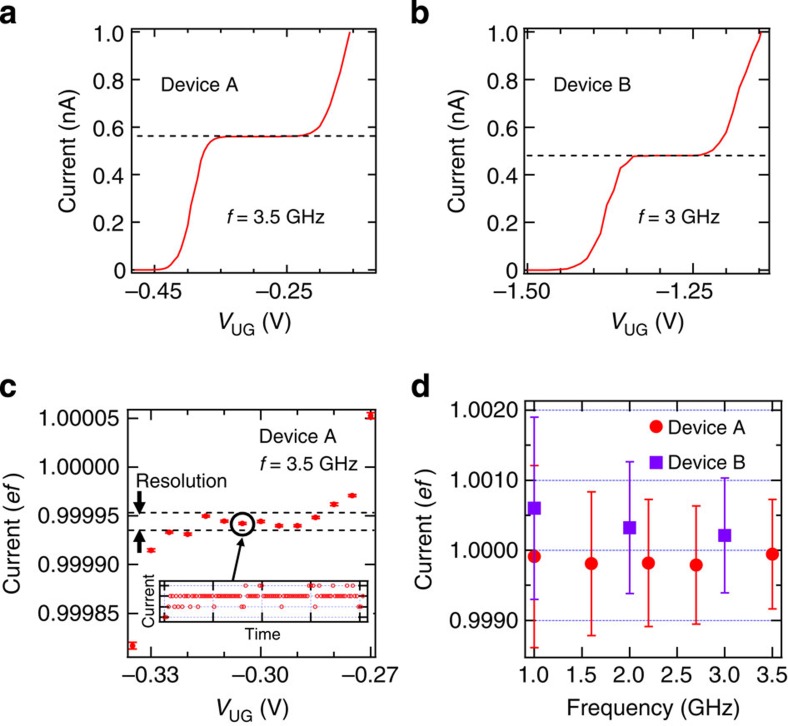
High-speed trap-mediated transfer. (**a**) Current as a function of *V*_UG_ at *f*=3.5 GHz in device A, where *V*_G2_=−0.7 V, *V*_BG_=10 V, the output power and offset of the sinusoidal signal are 18 dBm (the amplitude of about 5 V) and −1 V, respectively, and *T*=17 K. (**b**) Current as a function of *V*_UG_ at *f*=3 GHz in device B, where *V*_G2_=−1 V, *V*_BG_=12 V, *V*_ON_=0.8 V, *V*_OFF_=−3.2 V, *τ*_SW_~100 ps, *τ*_ON_=*τ*_OFF_ and *T*=17 K. (**c**) Current normalized by *ef* with high-resolution measurements in device A at 3.5 GHz, where the voltage conditions are the same as those in **a**. The error bars are the random uncertainty *U*_R_. These data show an excellent flatness of the 1*ef* plateau corresponding to the trap-mediated transfer. Inset: the typical raw current data, where the spacings between the adjacent horizontal and vertical dotted lines are the resolution of the measurement system (10 fA) and 50 s, respectively. The average of the raw data corresponds to the point indicated by a circle in the main panel. (**d**) Current normalized by *ef* as a function of frequency in devices A and B. The error bars are the total measurement uncertainty *U*_T_ discussed in Methods. Since the uncertainty of the measurement systems (systematic uncertainty *U*_S_) is dominant, the SE transfer accuracy is likely below the uncertainty (~10^−3^).

We here demonstrate high-speed operation of the trap-mediated transfer. Figure 5a,b shows the transfer current measured in devices A and B as a function of *V*_UG_ at frequencies of 3.5 and 3 GHz, respectively. The corresponding current levels are about 560 and 480 pA, which satisfy the current-level criterion for the current standards. Then, we measured the transfer current in more detail for device A as a function of *V*_UG_ (Fig. 5c), where the current is normalized by *ef* (we used *e*=1.602176565 × 10^−19^, which is the 2010 CODATA value^37^). Since we took many data points with a long integration time in this measurement (the inset in Fig. 5c shows one set of the raw data; see Methods), the random uncertainty *U*_R_ indicated by the error bars in Fig. 5c is sufficiently low compared with the resolution of the current meter (10 fA). Seven data points are within the measurement resolution, indicating the excellent flatness (better than 5 × 10^−5^) of the current plateau. Note that the absolute values in Fig. 5c are not known because the systematic uncertainty is much worse than *U*_R_ as discussed below.

To investigate the total measurement uncertainty, the systematic uncertainty *U*_S_ must be taken into account. We measured the transfer current at the flattest part of the 1*ef* plateau (indicated by a circle in Fig. 5c) as a function of frequency for device A and B (Fig. 5d), where the error bar is the total measurement uncertainty 
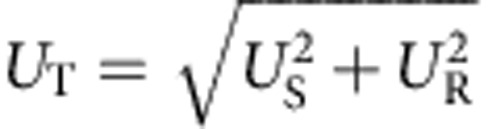
 and the level of *U*_R_ is the same as that in Fig. 5c (see Methods). Since *U*_T_/*ef* of about 7.8 × 10^−4^ for device A at 3.5 GHz and 8.2 × 10^−4^ for device B at 3 GHz are dominated by *U*_S_, the accuracy of the SE transfer should be better than these values. In a future experiment, we need a high-precision measurement using, for example, SE counting^11^ to estimate the true accuracy. Using *U*_T_ and equation (1), we estimated the lower bound of the capture and emission speeds, which is about 4–5 × 10^10^ s^−1^ (corresponding time scale is 20–25 ps), suggesting that high-speed capture and emission have been achieved.

The observation of the high-speed transfer for device B indicates that the performance of the device with a large activation energy (~37 meV) is stable. Thus, it is worth evaluating the order of the electric field at the trap level and whether the high-speed SE emission with the large *E*_act_ is reasonable. From a simple capacitance model with the device dimensions, we crudely estimated the electric field at the trap level for device B during 3-GHz operation, which is on the order of V μm^−1^. In addition, from the electric field-assisted emission theory with the Frenkel–Poole effect^38^, the theoretical emission rate from the trap level with *E*_act_ was also crudely estimated. The result was that the emission rate can be sufficiently high (even 10^10^ s^−1^ order) also with an electric field on the order of V μm^−1^. These estimations support the observation of the high-speed transfer.

## Discussion

We discuss the accuracy of the trap-mediated transfer in more detail. The possible error sources can be classified as follows: (i) missed transitions due to insufficient capture and emission speeds, (ii) leakage to the left lead during the rise of the barrier, (iii) other unexpected errors. First, we estimate the missed transitions. As discussed above, the capture and emission speeds are more than 4–5 × 10^10^ s^−1^ at more than 3 GHz. Since the speed is determined by the potential distribution, that is, the voltage conditions, the same speed can be achieved at a lower frequency, for example, 1 GHz. It is then possible to predict the missed transition rate at a lower frequency, corresponding to longer *τ*_ON_ and *τ*_OFF_ in equation (1). The predicted rate then approaches 1 × 10^−9^ at 1 GHz, which is sufficiently low. Second, we consider the leakage errors. We have estimated the activation energies to be 13 and 37 meV for devices A and B, respectively. From these values and equation (2), we estimated the relative leakage error rate (1−*P*) at a low temperature. At less than 10 and 22 K for devices A and B, respectively, the relative leakage error rate is less than 1 × 10^−8^. It should be noted that the relative leakage error rate decreases with decreasing *τ*_SW_ (typical data are shown in Supplementary Fig. 3). Finally, we discuss the unexpected errors. Although the missed transition and leakage error can be sufficiently low, the unexpected errors may emerge when we measure the transfer accuracy with a higher precision. One possibility is nonadiabatic excitation^39^, where nonadiabatic transitions of an SE from a ground state to excited states occur during the fast rise of the barrier, leading to another leakage error to the lead. Another possibility is the contribution of other trap levels. If an additional trap level that has a much larger or smaller activation energy exists, it is possible that another SE with an undetectable low current level could be transferred, which could limit the transfer accuracy. Thus, we need high-precision measurements to investigate the true accuracy in future work.

Finally, we discuss the origin of the trap level and the device yield. The trap level in the present device most likely originates from an interface trap between Si and SiO_2_ because we used the conventional Si-MOSFET fabrication technique and the Si nanowire was undoped. Considering the typical density of interface traps in Si MOSFETs (~10^10^ cm^−2^) (ref. 29), the average number of interface trap levels under G1 (~10^−11^ cm^2^) should be about 0.1. Therefore, we needed to test many devices to find those having excellent SE transfer characteristics. Some devices show the high-speed transfer (data are shown in Supplementary Fig. 4), but the yield of such devices is low (~3%) because of the random position of the trap levels. However, it is not so difficult to find high-speed SE transfer devices because we can fabricate many devices on an Si wafer and can test about a hundred devices per day using a low-temperature probe station. In addition, we have reported the excellent stability of the tunable-barrier Si devices^40^, indicating their suitability of the metrological application. Nevertheless, it would be beneficial to use position-controlled localized states instead of randomly positioned trap levels. One promising way to do this is to utilize dopants with position-control techniques such as scanning tunnelling microscope patterning^41^ or implantation with nano-apertures^24^.

In summary, we have demonstrated SE transfer using a single localized trap level in Si MOSFETs. By analysing the pulse-amplitude and temperature dependence of the transfer current, we have elucidated the transfer mechanism. The device with the optimally positioned trap level allows us to generate a sufficiently large current with high-frequency operation of up to 3.5 GHz at 17 K without a magnetic field, where the transfer accuracy of about 10^−3^ is limited by the total measurement uncertainty. In addition, the time-dependence experiment revealed that the rates of capture/emission to/from the single-trap level is well controlled electrically, which opens a pathway to utilizing a localized state, such as a single dopant/trap, for future metrology and electronics based on single-charge manipulation.

## Methods

### Device fabrication

The device fabrication process is as follows. First, an Si nanowire was formed on a 400-nm-thick buried oxide by using electron beam lithography, followed by thermal oxidation for the formation of a gate oxide. Next, two polycrystalline-Si lower gates (G1, G2) were formed on the nanowire. Then, an interlayer oxide was grown by chemical vapour deposition. After that, the entire region in the image shown in Fig. 1b was covered with a polycrystalline-Si UG. Finally, *n*-type leads were formed by ion implantation with the UG used as a mask. The device dimensions are summarized in Supplementary Table 1.

### Measurement systems

The measurement temperature was 17 K, except for the temperature-dependence experiment (Fig. 4). We used an Agilent B1500A semiconductor device parameter analyzer to apply DC voltages and measure a current. The traceable calibration of the B1500A was performed on 15 January 2013 and 7 March 2014 with 1 year expiration date. All data were measured within the expiration date. The voltage pulses were applied from an Agilent 81160A pulse generator (*f*≤330 MHz), which was used in Figs 1, 2, 3, 4, or an Agilent 81134A pulse pattern generator (15 MHz ≤ *f* ≤3.3 GHz), which was used for device B in Fig. 5. The sinusoidal voltage was applied from an Agilent E8257D analogue signal generator (250 kHz ≤ *f* ≤20 GHz), which was used for device A in Fig. 5 to access frequencies above 3.3 GHz, where we used a KEYCOM bias tee (KDC-H5004G) to apply an offset voltage. Since a large amplitude is important for the operation of the high-speed trap-mediated transfer, using the sinusoidal voltage is not a problem. For the high-frequency pulse measurement (device B in Fig. 5d), we used a CERNEX broadband medium power amplifier to increase the amplitude of the voltage pulses.

### High-resolution measurement and uncertainty

For the measurements in Fig. 5c,d, we used the longest integration time (two second) of the B1500A for one data point and 101 data points were averaged. The random uncertainty *U*_R_ is determined by the s.d. of the mean value of the current (the s.d. of the 101 data points divided by 
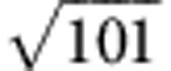
). When we measure the current using the B1500A (the measured value is *I*_m_) in a measurement range of 1 nA with the built-in high-resolution analogue-digital converter, the measurement resolution is 10 fA (corresponding to the spacing between the adjacent horizontal dotted lines in the inset in Fig. 5c) and the true value exists within *I*_m_±Δ=*I*_m_±[*I*_m_ × 10^−3^+2 × 10^−13^+*V*_0_ × 10^−15^], where *V*_0_ is the applied voltage to the measurement terminal. This uncertainty is guaranteed by the traceable calibration. Since *V*_0_ is much less than 1 V, we neglected the third term. In addition, we assumed a uniform distribution of the existence probability of the true value between *I*_m_+Δ and *I*_m_−Δ, which is the worst case. Since the systematic uncertainty is dominated by Δ, we calculated 
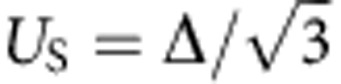
 (the s.d. of the rectangular probability distribution). Note that since *I*_m_~*ef* in our measurements but the last two terms of Δ are fixed values, *U*_S_ depends on the frequency. As a result, we obtained the total measurement uncertainty 
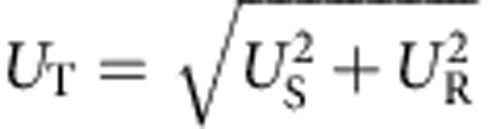
, where the confidence level is about 68 % (1*σ*). In Fig. 5d, we plotted *U*_T_ as the error bars.

## Author contributions

G.Y. conceived the experiments, performed the measurements, analysed the data and prepared the manuscript. K.N. and A.F. fabricated the devices. All authors discussed the results and commented on the manuscript. A.F. supervised the project.

## Additional information

**How to cite this article:** Yamahata, G. *et al*. Gigahertz single-trap electron pumps in silicon. *Nat. Commun.* 5:5038 doi: 10.1038/ncomms6038 (2014).

## Supplementary Material

Supplementary Information Supplementary Figures 1-4, Supplementary Table 1, Supplementary Notes 1-2 and Supplementary References.
