# Supplementary material for: Gigahertz single-trap electron pumps in silicon
Source: Nat Commun. 2014 Oct 6;5:5038. doi: 10.1038/ncomms6038 (PMC4205845; doi:10.1038/ncomms6038)
Supplement: Supplementary Information — Supplementary Figures 1-4, Supplementary Table 1, Supplementary Notes 1-2 and Supplementary References. [file ncomms6038-s1.pdf]

Supplementary Figures

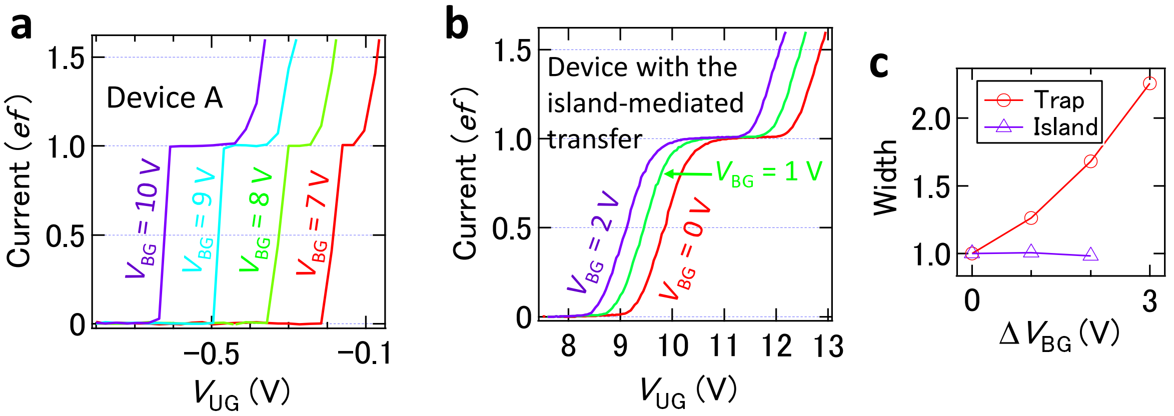

Supplementary Figure 1. **Backgate dependence of trap-mediated transfer.** **a**, Current normalized by ef as a function of V_{UG} with changing V_{BG} from 7 to 10 V in device A, where $f = 10$ MHz, $V_{G2} = -0.3$ V, $V_{ON} = 0.25$ V, $V_{OFF} = -1.5$ V, $\tau_{SW} = 1$ ns, $\tau_{ON} = \tau_{OFF}$, and $T = 17$ K. **b**, Current normalized by ef as a function of V_{UG} with changing V_{BG} from 0 to 2 V in a device without the trap levels, where $f = 10$ MHz, $V_{G2} = -0.8$ V, $V_{ON} = 0.5$ V, $V_{OFF} = -3$ V, $\tau_{SW} = 2$ ns, $\tau_{ON} = \tau_{OFF}$, and $T = 24$ K. **c**, Width of the $1ef$ plateau normalized by that at $V_{BG} = 7$ V for device A (trap) and that at $V_{BG} = 0$ V for the device shown in **b** (island) as a function of the change in V_{BG}.

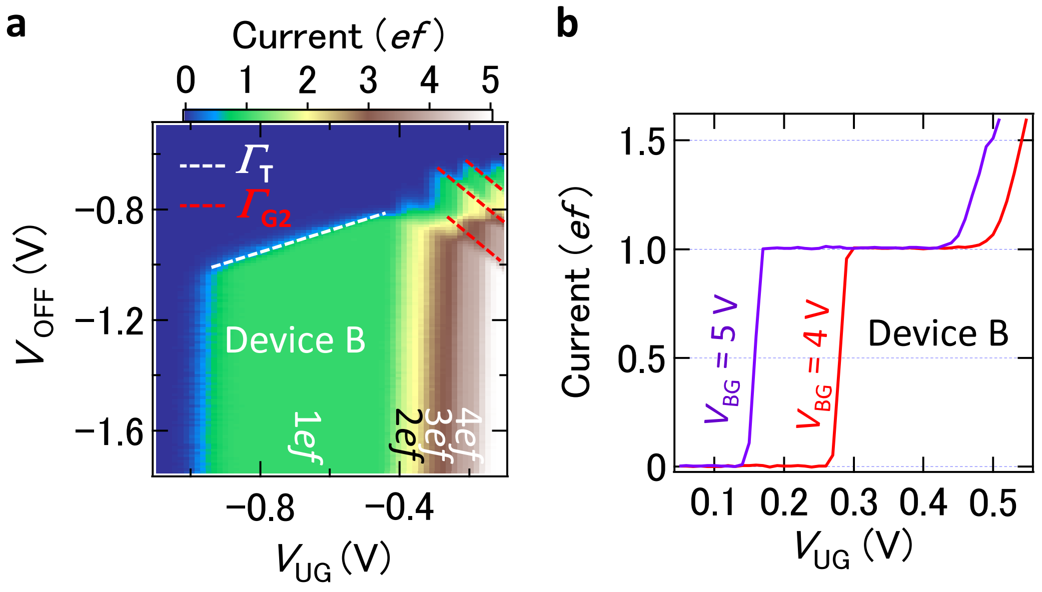

Supplementary Figure 2. **Trap-mediated transfer in device B.** **a**, Two-dimensional plot of the current normalized by ef as a function of V_{UG} and V_{OFF} in device B, where $f = 10$ MHz, $V_{\mathrm{G2}} = -0.65$ V, $V_{\mathrm{BG}} = 20$ V, $V_{\mathrm{ON}} = 0.75$ V, $\tau_{\mathrm{SW}} = 1$ ns, $\tau_{\mathrm{ON}} = \tau_{\mathrm{OFF}}$, and $T = 17$ K. As discussed in the main text (Fig. 2a), the positive slope of the threshold voltage (white dashed line) indicates the trap-mediated transfer. **b**, Current normalized by ef as a function of V_{UG} with changing V_{BG} from 4 to 5 V in device B, where $f = 10$ MHz, $V_{\mathrm{G2}} = -0.8$ V, $V_{\mathrm{ON}} = 0.5$ V, $V_{\mathrm{OFF}} = -3$ V, $\tau_{\mathrm{SW}} = 1$ ns, $\tau_{\mathrm{ON}} = \tau_{\mathrm{OFF}}$, and $T = 17$ K. We observed the width modulation of the $1ef$ plateau by V_{BG}, where the width ratio between the two data is about 1.3. The ratio is similar to that shown in Supplementary Figure 1b. Thus, we concluded that the $1ef$ plateau in device B corresponds to the trap-mediated transfer.

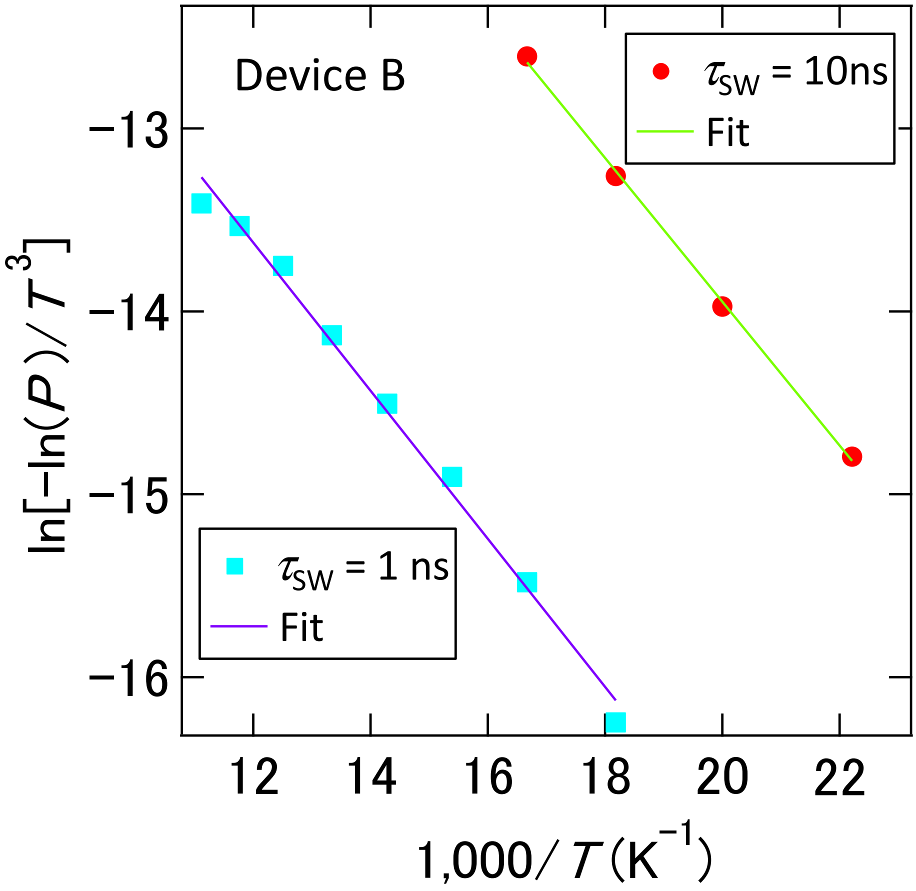

Supplementary Figure 3. **τ_{SW} effect on leakage error.** $\ln[-\ln(P)/T^3]$, where P is the transfer probability, as a function of inverse of temperature in device B. The measurement condition is as follows: $f = 10$ MHz, $V_{UG} = 0.16$ V, $V_{G2} = -1$ V, $V_{BG} = 5$ V, $V_{ON} = 0.5$ V, $V_{OFF} = -2.5$ V, $\tau_{SW} = 10$ ns (red dots) or 1 ns (blue squares), and $\tau_{ON} = \tau_{OFF}$. The green and purple lines are linear fits to the data. As expected, the slope of the linear fit, which corresponds to the activation energy, is almost the same between the two sets of data, but the y-intercept corresponding to τ_{SW} is different. At $\tau_{SW} = 10$ ns, the theoretically predicted relative leakage error rate is on the order of 10^{-7} at $T = 22$ K, which is one order of magnitude worse than that at $\tau_{SW} = 1$ ns. This indicates that the relative leakage error rate becomes lower with increasing operating speed.

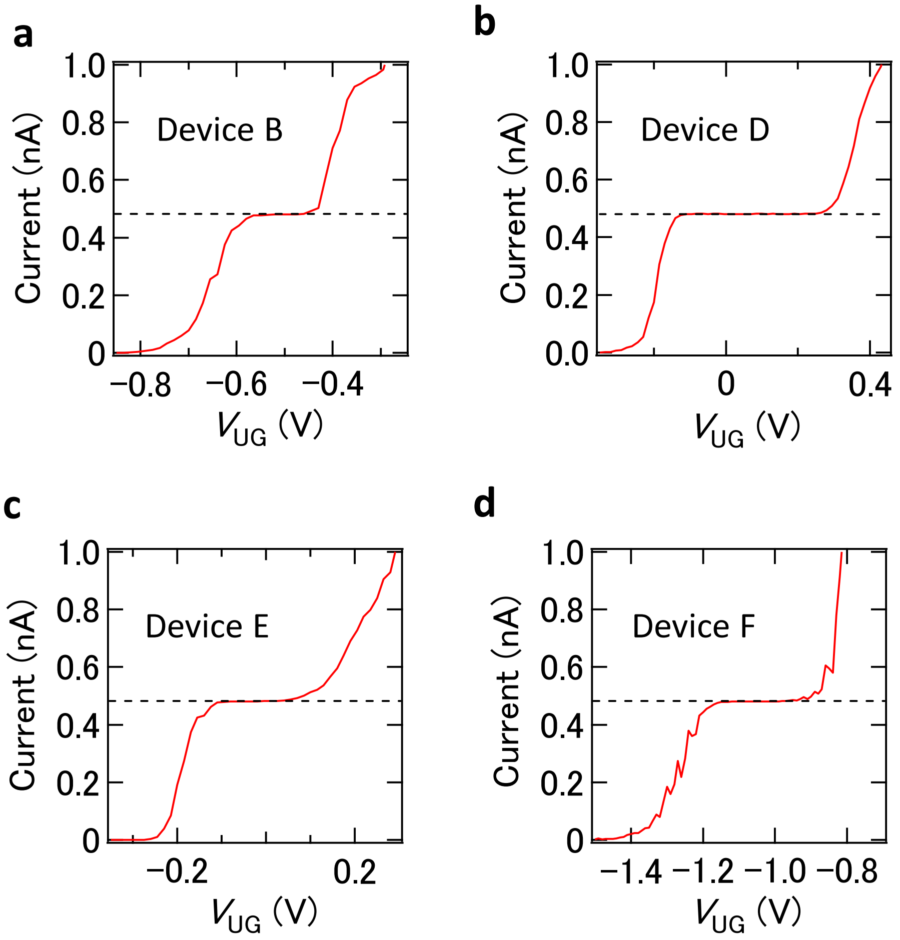

Supplementary Figure 4. **Four devices operating at 3 GHz.** **a-d**, Current as a function of V_{UG} at 3 GHz in four different devices. To find them, we tested 152 devices (yield $\sim$ 3%), suggesting the physics of the trap-mediated transfer is rather simple and guarantees the capability to transfer SEs with high speed. For all devices, $V_{\text{G2}} = -1$ V, $\tau_{\text{SW}} \sim 100$ ps, $\tau_{\text{ON}} = \tau_{\text{ON}}$, and $T = 17$ K. For device B (**a**), $V_{\text{BG}} = 12$ V, $V_{\text{ON}} \sim 3$ V, and $V_{\text{OFF}} \sim -5.2$ V. For device D (**b**), $V_{\text{BG}} = 7$ V, $V_{\text{ON}} \sim 3.3$ V, and $V_{\text{OFF}} \sim -5.5$ V. For device E (**c**), $V_{\text{BG}} = 5$ V, $V_{\text{ON}} \sim 3$ V, and $V_{\text{OFF}} \sim -5.2$ V. For device F (**d**), $V_{\text{BG}} = 20$ V, $V_{\text{ON}} \sim 3.2$ V, and $V_{\text{OFF}} \sim -5.3$ V.

Supplementary Table

Device	Gate length (G1, G2)	Gap between G1 and G2	Si-wire width	Si-wire thickness	SiO_2 thickness under G1	SiO_2 thickness under UG
A	∼50 nm	∼130 nm	∼30 nm	∼20 nm	∼30 nm	∼80 nm
B	∼20 nm	∼130 nm	∼30 nm	∼20 nm	∼30 nm	∼80 nm
C	∼20 nm	∼130 nm	∼50 nm	∼20 nm	∼30 nm	∼80 nm

Supplementary Table 1. **Dimensions of the devices shown in the main text.**

Supplementary Note 1

As backgate voltage V_{BG} increases, the width of the $1ef$ plateau in device A becomes large (Supplementary Figure 1a). This is because the spacial position of the trap level is different from that of the island[1]. The dominant effect should be the screening of the V_{UG} effect at the trap level by G1, because the position of the trap level should be under G1. This leads to the smaller capacitive coupling ratio of the UG to the backgate ($C_{\mathrm{UG}}/C_{\mathrm{BG}}$) for the trap level than for the island. As a result, when V_{BG} increases, the threshold voltage shift is larger for the trap-mediated transfer than for the island-mediated transfer. When the $1ef$ and $2ef$ plateaus are the trap-mediated and island-mediated transfers, respectively, the different threshold voltage shift results in the widening of the $1ef$ plateau, which is observed in device A. On the other hand, in the device without trap levels, the width of the $1ef$ plateau is almost constant (Supplementary Figure 1b) because all the plateaus originate from the island-mediated transfer. To clearly show the difference between the two devices, we plotted the $1ef$ plateau width normalized by that at the smallest V_{BG} as a function of change in V_{BG} (Supplementary Figure 1c). Thus, we conclude that the modulation of the plateau width is one of the signatures of the trap-mediated transfer.

Supplementary Note 2

To estimate Γ_{E} and Γ_{C}, we need to observe a clear $1ef$ plateau with sufficiently high V_{ON} and low V_{OFF}. The observation of the $1ef$ plateau (Figs. 1e-1g in the main text) indicates that, within the uncertainty of the measurement systems ($\sim 10^{-3}$), we can assume that (i) the threshold voltage of the island-mediated transfer is far from that of the trap-mediated transfer, (ii) the leakage error during the rise of the barrier is sufficiently small, and (iii) the emission to the left lead is sufficiently small during τ_{OFF}. Under these assumptions, we derive an analytical formula of the capture and escape dynamics of the trap-mediated transfer.

Since the emission of an SE in the trap-mediated transfer should be determined by only Γ_{E}, the emission probability $P_{\mathrm{E}}(t)$ is determined from the rate equation of the SE emission, $dP_{\mathrm{E}}(t)/dt = \Gamma_{\mathrm{E}} \cdot [1 - P_{\mathrm{E}}(t)]$. By integrating it with the condition $P_{\mathrm{E}}(0) = 0$ ($t = 0$ just after

the pulse voltage becomes the off-state),

$$P_{\mathrm{E}}(t) = 1 - \exp(-\mathit{\Gamma}_{\mathrm{E}} \cdot t). \tag{1}$$

In contrast, the capture of an SE in the trap-mediated transfer may be determined not only by $\mathit{\Gamma}_{\mathrm{C}}$ but also by escape rate $\mathit{\Gamma}_{\mathrm{OUT}}$ from the trap level to the left lead during τ_{ON}, which is not depicted in Fig. 1d in the main text. Therefore, the rate equation for the capture probability $P_{\mathrm{C}}(t)$ is

$$\frac{dP_{\mathrm{C}}(t)}{dt} = -\mathit{\Gamma}_{\mathrm{OUT}} \cdot P_{\mathrm{C}}(t) + \mathit{\Gamma}_{\mathrm{C}} \cdot [1 - P_{\mathrm{C}}(t)]\,. \tag{2}$$

The analytical solution of the differential equation with the condition $P_{\mathrm{C}}(0) = 0$ ($t = 0$ just after the pulse voltage becomes the on-state) is

$$P_{\mathrm{C}}(t) = \frac{\mathit{\Gamma}_{\mathrm{C}}}{\mathit{\Gamma}_{\mathrm{C}} + \mathit{\Gamma}_{\mathrm{OUT}}} \cdot \{1 - \exp\left[-\left(\mathit{\Gamma}_{\mathrm{C}} + \mathit{\Gamma}_{\mathrm{OUT}}\right) \cdot t\right]\}\,. \tag{3}$$

However, we can simplify it as follows. When we apply large gate voltages (V_{UG} or V_{ON}), the trap level should be sufficiently lower than the Fermi level in the lead, where $\mathit{\Gamma}_{\mathrm{C}}$ should be much greater than $\mathit{\Gamma}_{\mathrm{OUT}}$. We can estimate a typical value of $\mathit{\Gamma}_{\mathrm{C}}/\mathit{\Gamma}_{\mathrm{OUT}}$ in such a case by using activation energy E_{act}. As a typical value of E_{act}, we used 13 meV extracted from the temperature-dependence measurement in Fig. 4b in the main text. At $T = 17$ K, when we assume a thermal emission of an SE and a detailed balance,

$$\mathit{\Gamma}_{\mathrm{C}}/\mathit{\Gamma}_{\mathrm{OUT}} \sim \exp\left(\frac{E_{\mathrm{act}}}{k_{\mathrm{B}}T}\right) \sim 7 \times 10^{4}, \tag{4}$$

where k_{B} is the Boltzmann constant. This indicates that the relative error rate related to $\mathit{\Gamma}_{\mathrm{OUT}}$ should be sufficiently low within the uncertainty of the measurement systems ($\sim 10^{-3}$). In this case, Supplementary Equation 3 is simplified as

$$\tilde{P}_{\mathrm{C}}(t) = 1 - \exp(-\mathit{\Gamma}_{\mathrm{C}} \cdot t). \tag{5}$$

Therefore, we used Supplementary Equation 5 as $P_{\mathrm{C}}(t)$ in the main text. As a result, the transfer probability P of an SE is given by

$$\begin{aligned} P &= P_{\mathrm{C}}(\tau_{\mathrm{on}}) \cdot P_{\mathrm{E}}(\tau_{\mathrm{off}}) \\ &= [1 - \exp(-\mathit{\Gamma}_{\mathrm{C}} \cdot \tau_{\mathrm{on}})] \cdot [1 - \exp(-\mathit{\Gamma}_{\mathrm{E}} \cdot \tau_{\mathrm{off}})]\,. \end{aligned} \tag{6}$$

Supplementary References

[1] Yamahata, G., Nishiguchi, K. & Fujiwara, A. Accuracy evaluation of single-electron shuttle transfer in Si nanowire metal-oxide-semiconductor field-effect transistors. *Appl. Phys. Lett.* **98**, 222104 (2011).
